# Influence of combined action observation and motor imagery of walking on lower limb reflex modulation in patients after stroke–preliminary results

**DOI:** 10.1186/s13104-022-06057-5

**Published:** 2022-05-13

**Authors:** Frank Behrendt, Monika Le-Minh, Corina Schuster-Amft

**Affiliations:** 1 Research Department, Reha Rheinfelden, Rheinfelden, Switzerland; 2 Department of Engineering and Information Technology, Bern University of Applied Sciences, Bern, Switzerland; 3 Department of Health, Bern University of Applied Sciences, Bern, Switzerland; 4 Department of Sport, Exercise and Health, University of Basel, Basel, Switzerland

**Keywords:** Action observation, Motor imagery, Reflex responses, Stroke, Walking

## Abstract

**Objective:**

The combined use of action observation and motor imagery (AOMI) is a promising technique in neurorehabilitation that can be usefully applied in addition to conventional forms of therapy. Previous studies with healthy participants showed that the mere passive observation of walking results in a phase-dependent reflex modulation in the *tibialis anterior* muscle that resembles the pattern occurring when walking. In patients after stroke, a similar reflex modulation was found in several lower limb muscles during the real execution of walking, but responses were blunted. To clarify whether and how lower limb reflex responses are also modulated in such patients during the combined synchronous observation and imagery of walking, medium-latency cutaneous reflexes from the *tibialis anterior* muscle were measured. We compared the reflex responses of seven patients after stroke during the AOMI of walking from two different conditions: (a) elicited during the end stance phase and (b) during the end swing phase, both normalized to a baseline condition.

**Results:**

So far, using the identical methodological set-up as in our study with healthy individuals, we could not find any noteworthy reflex response modulation. *The study was registered with the German Clinical Trials Register (DRKS00028255).*

*Trial registration* : The study was registered with the German Clinical Trials Register: DRKS00028255.

## Introduction

For patients with neurological disorders, action observation (AO) and motor imagery (MI) can be useful and safe tools [1–15]. Compared with either AO or MI applied alone, previous studies with healthy participants revealed a stronger effect when action observation is combined and simultaneously performed with motor imagery (AOMI) [16–23]. There is also first evidence of a beneficial effect of synchronous AOMI in chronic stroke rehabilitation [24].

During AO, the actual execution or during MI of an action, similar premotor and parietal neural structures in the perceiver are recruited [25, 26], thus sharing cortical networks [27]. While this effect has been well studied at the cortical level, we reported on the influence of AO at a more peripheral level of the motor system in individuals without impairment [28, 29]. The phase-dependent reflex behaviour in certain leg muscles that occurs as a result of cutaneous electrical stimulation during walking [30] could be found in individuals who were only passively sitting and observing the walking movement of another person [28]. Since psychophysiological changes in the peripheral and central systems can occur in a seated viewer compared to an upright observer [31], this result was not necessarily to be expected. In this respect, the seated posture certainly has a strong influence on the accessibility of the motor representation.

However, the data demonstrated that healthy individuals in a seated position watching another person walking, recreate a mental image of the observed gait which they reproduce concurrently in lower limb EMG recordings [32]. As an altered reflex response behaviour during real walking was reported in stroke survivors [33], the aim of the current project was to find out whether modulated, phase-dependent reflex responses during synchronous AOMI [24] of walking could be found in stroke survivors. To the best of our knowledge, no study has yet been conducted on cutaneous reflex responses in post-stroke patients during action observation of walking alone or combined with motor imagery. We hypothesised that there would be a modulation of the reflex responses in the patients according to the gait phases, but that it would be somewhat less pronounced than in the group of healthy individuals in our previous study [28].

## Main text

### Study population

Data have been collected so far from seven stroke patients (two women) during their stay in a rehabilitation clinic in Rheinfelden, Switzerland. Patients were enrolled between September 2019 and February 2020. Continuation of the recruitment process was not possible due to the Corona-related restrictions and the subsequent termination of the project. The mean age of the patients (2 females) was 57.29 ± 16.41 years (SD) ranging 26 to 71 years with an average time since the stroke of 42.57 ± 51.14 months (Table 1). Inpatients with a diagnosis of ischaemic or haemorrhagic stroke could be included if they were able to sit independently and scored higher than 19 in the Montreal Cognitive Assessment [34]. They further needed to satisfactorily score in two out of three of the following motor imagery ability instruments: (a) Kinesthetic and Visual Imagery Questionnaire score of 30/50 [35], (b) Mental rotation > 75% [36], and (c) Mental chronometry ratio of 1 ± 0.25 [35]. Exclusion criteria comprised a history of multiple strokes, visual impairment, epilepsy seizures in the last year, other neurological, metabolic or mental disorders and a pacemaker.

**Table 1:** Patient characteristics and outcome scores

ID	Age range	Month post-stroke	Type and site of lesion	KVIQ	MR	MC	FAC	NRR in % during stance phase	NRR in % during swing phase
1	65–69	3	I, right, posterior CR	40	32	1.23	5	114.7	93.1
2	65–69	14	I, right, middle CR	50	31	1.02	5	112.8	122.9
3	70–74	123	H, right, thalamus and IC	33	27	0.49	4	93.7	99.7
4	25–29	1	I, left, middle CR	40	25	0.94	5	112	110.1
5	45–49	26	I, right, middle CR	28	33	1.00	5	67.9	59.2
6	55–59	22	H, right BG	43	27	0.89	5	77.2	78.3
7	70–74	109	I, right BG	22	27	0.87	5	113.6	110.7

*BG* basal ganglia, *CR* cerebral artery, *FAC* functional ambulation category, *H* haemorrhagic, *I* ischemic, *IC* internal capsule, *KVIQ-10* kinaesthetic and visual imagery questionnaire, *MC* mental chronometry, *MR* mental rotation, *NRR* normalized reflex response

### Set-up

We used the same set-up, procedure and analysis as in our study with healthy participants [28]. Cutaneous electric stimulation on the foot was applied which can elicit reflex responses in several leg muscles [37]. These responses, starting from about 75–80 ms are known as P2 (medium latency) responses (see Duysens et al. 2004 for a comprehensive description) [38]. Exemplary reflex traces can be found in our previous report.

For displaying human walking, point-light biological motion [39] was used, which was based on 3-D recordings of a real walking movement on a treadmill. The visual stimulus presented a slightly oblique back-view of walking (slanted to the right by 13°) which measured 6 × 15 cm on the 22″ TFT display. To ensure that the observers perceived the stimulus as a back-view, the markers were occluded when covered by body parts. This was originally chosen to support a first-person image of the walking movement. The notebook used to present the visual stimulus also triggered the electrical stimulation (Digitimer DS7A, Welwyn Garden City, UK). The stimulation electrode (Axelgaard, Fallbrook, CA, USA) was placed at the medial side of the right ankle, where the posterior tibial nerve is closest to the skin [40]. The EMG of the right *tibialis anterior* muscle was recorded at 2000 Hz (Myon, myon AG, Schwarzenberg, Switzerland) using bipolar, amplified surface electrodes with an inter-electrode distance of 21 mm. The surface electrodes were placed at 1/3 on the line between the tip of the fibula and the tip of the medial malleolus according to the SENIAM-guidelines [41]. The recorded signal was band-pass filtered (30–300 Hz), rectified and averaged using Matlab (Mathworks, Natick, MA, USA).

We used the identical set-up with all patients irrespective of the side of lesion. It was originally planned to conduct a subgroup analysis once the sample size was sufficient. However, there is evidence that transcranial magnetic stimulation-evoked potentials are higher in stroke patients on both the affected and unaffected sides during AO [42].

### Procedure

All patients were invited for a single measurement session. After completion of initial measurements to verify eligibility, patients were asked to sit comfortably on a normal chair in front of the 21.5" TFT monitor that displayed the point-light stimuli and to watch them for 5 min. to get accustomed to the task before the experiment started. They were instructed to remain completely relaxed while observing the visual stimuli attentively and simultaneously imagining the synchronous execution of the observed walking movement [24]. The MI part of the task was performed from an internal perspective with a kinaesthetic mode [43]. The experiment consisted of 39 trials of the visual walking stimulus (26 trials) and a baseline condition (13 trials). In the baseline condition, each of the individual white dots that made up the point-light figure, performed the same movements, i.e. movement trajectories, but in a different, random position on the screen. Thus, no human figure or biological movement could be recognized. All visual trials were presented in random order, each lasting 10 s with a 10-s pause in between, during which a blank screen was shown. The patients were not told that the stimulation was timed with respect to the walking phase. For the presented walking this was either at the end of a stance phase (13 trials) or at the end of a swing phase (13 trials), triggered by the presentation software which was an inhouse programmed application (MotionViewer) using XCode 3.1 and OpenGL.

In order to reliably obtain reflex responses during the experiment, we followed the usual procedure during the preparation phase [34]. During quiet standing, the motor threshold was determined by gradually increasing the stimulus intensity until a visible muscle contraction was elicited in the *abductor hallucis* which was possible for all included patients. The stimulation intensity was set at 1.5 times the motor threshold.

### Analysis

Reflex responses elicited by the electrical stimulation [37] were analysed for the three conditions: (a) stance phase, (b) swing phase and (c) baseline by calculating the integral of the root mean square (RMS) of the EMG signal of the time window between 80 and 130 ms from the beginning of the electrical stimulation [44]. For each subject the mean reflex responses for the two walking phases were normalized to the baseline condition. The reflex increase or decrease was defined as the percentage change of the RMS. The data analysis was kept analogous to the established methods for active walking [30, 45, 46].

### Results

**Fig. 1:**
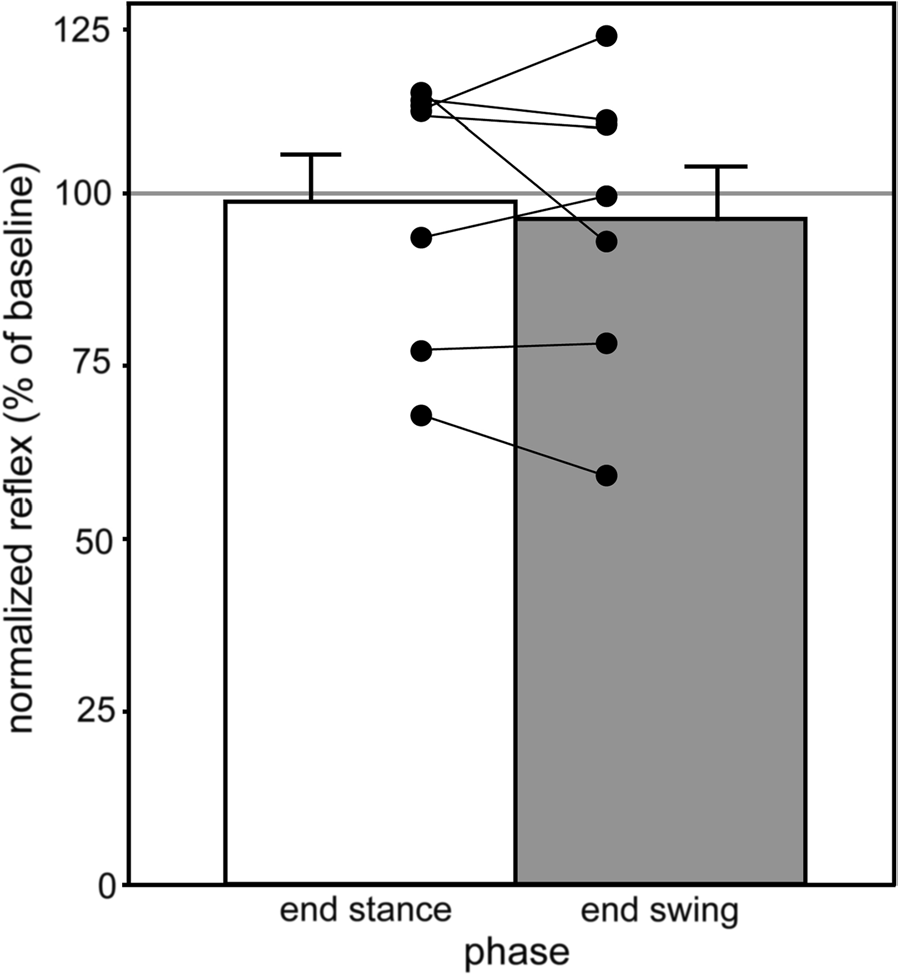
Mean (RMS after rectification) normalized responses of seven stroke patients with standard errors and individual, normalized values. Horizontal grey line: baseline/control condition

On average, the normalized reflex responses were reduced by 1.2% (± 19.5% standard deviation) for the end stance phase condition, and also reduced by 3.7% (± 21.7% standard deviation) for the end swing phase (Table 1). The preliminary, paired t-test performed on the normalized responses (Fig. 1) despite the small sample size indicated that there was no modulation (t(6) = 0.65, P = 0.542, d = 0.246). Thus, the evoked EMG responses between the observed and imagined end stance phase and end swing phase did not differ in the stroke patients. In none of the patients, as in our previous study [28], could background muscular activity in the right *tibialis anterior muscle* be detected, which theoretically could have led to exclusion.

### Discussion

There is a huge interest in exploiting the positive effect of the observation and mental recreation of motor activities to improve motor function after stroke. These treatment approaches have yielded promising results, suggesting that AO and MI represent valuable additional interventions [10, 14]. However, it might be helpful to better identify those patients for whom AO and MI would be particularly beneficial as for instance the length of time since the stroke and the extent of physical disengagement could play a role. Therefore, the current study aims to understand whether stroke patients, in general, show the same reaction on the muscular level during AO combined with MI.

The preliminary data we collected so far differed from that of healthy, unaffected individuals, as the reflex responses of the stroke survivors were rather similar for all conditions revealing no changed reflex gain according to the phase of the observed and imagined gait cycle. In our earlier study in healthy individuals [28], the difference between the two gait phases was significant, with a modulation difference of more than 30% whereas in the current study we found a difference of only 2.5% in the rather heterogeneous data of the PaS. This deviation occurred even though all stroke patients were able to walk. Zehr and colleagues comprehensively studied the reflex network in stroke survivors during real walking [33]. They examined reflex responses in the more and less affected leg evoked by stimulation at the ankle and wrist during real walking in chronic stroke and found a phase-dependent modulation of cutaneous reflexes in stroke survivors and healthy individuals. Interestingly, responses were blunted in stroke survivors but still present.

Using functional magnetic resonance imaging it could be shown that during AO, as in healthy individuals, a neuronal activation can be found that overlaps with the activation during motor execution for example in premotor, motor and parietal cortical areas [47]. Brunner et al. included subacute stroke survivors who revealed an even increasing activation response in parallel with an improvement of their motor function and according to clinical recovery. This raises the question of whether and how the descending processing might have been affected in the stroke survivors of the current study. In a study investigating the effect of motor imagery on the F-wave elicited from a paretic muscle as a result of stroke, it was suggested that MI can support the process of restoring motor neuron excitability, which is depressed after stroke [48]. Such peripheral changes after stroke might have resulted in an altered modulation of activity in the current study, but can also be positively influenced by AOMI. A further possibility would be to examine corticospinal connections, in particular by measuring the high-frequency, descending waves that occur with transcranial magnetic stimulation, i.e. the D and I-wave [49, 50]. As the I-wave requires the integrity of the cortical grey matter [51], a disrupted grey and white matter network after stroke [52] may also influence a modulation on this level during AOMI.

A larger database would be helpful to determine the influence of the extent of disengagement from physical activity or the duration since stroke. A specification for the current recommendation on the use of AO and MI or AOMI in this regard as an adjunct to physical therapies cannot be made yet. In order to possibly achieve a stronger effect for instance also on lower limb function in gait rehabilitation, it might be helpful to evaluate the combination of AO/MI with repetitive transcranial magnetic stimulation. With regard to the upper extremities, promising results have been reported [53].

## Limitations

- Incongruence during motor imagery may have been enhanced as the patients viewed a model walking from a third person perspective but were asked to imagine the movement from a first person, internal perspective.
- Patients were tested for their motor imagery ability in general, but not explicitly asked for the vividness of their image of walking.
- More data is needed to verify the preliminary results and to be able to draw a statistically robust conclusion. A differentiated recommendation which would be transferable to a larger population cannot yet be given.
- The data collected so far reveal a rather large heterogeneity.

## Data Availability

The datasets generated and analysed during the current study are available from the corresponding author on reasonable request.

## Abbreviations

AO: Action observation
AOMI: Combined action observation and motor imagery
MI: Motor imagery
RMS: Root mean square
PaS: Patients after stroke
